# Feeding Pathway for Children on High Flow Nasal Cannula Decreases Time to Enteral Nutrition

**DOI:** 10.1097/pq9.0000000000000608

**Published:** 2022-12-07

**Authors:** Sara H. Soshnick, Gabriella S. Mark, Jacqueline Weingarten-Arams, Ying Chuu, Swati Chandhoke, Shivanand S. Medar, Kaitlyn Philips, Gina N. Cassel-Choudhury

**Affiliations:** From * Department of Pediatrics, Division of Critical Care, The Children’s Hospital at Montefiore, Bronx, N.Y.; † Department of Pediatrics, Division of Pediatric Critical Care Medicine, Columbia University Medical Center, Morgan Stanley Children’s Hospital of New York, New York, N.Y.; ‡ Department of Pediatrics, Division of Critical Care Medicine, Children’s Healthcare of Atlanta at Egleston, Atlanta, Ga.; § Department of Pediatrics, Division of Pediatric Hospital Medicine, The Children’s Hospital at Montefiore, Bronx, N.Y.

## Abstract

High Flow Nasal Cannula (HFNC) is commonly used for children with respiratory failure, yet no standardized guidelines exist on how to initiate, escalate, and maintain enteral nutrition (EN) for these patients. EN in critically ill children is associated with decreased hospital length of stay, decreased ventilator days, and fewer acquired infections. We aimed to decrease the mean time to EN initiation by 50% after the start of HFNC in 6 months.

**Methods:**

This quality improvement project used the Model for Improvement to inform interventions. A multidisciplinary team created an EN pathway for critically ill patients on HFNC. We conducted Plan-Do-Study-Act cycles related to implementing a standardized pathway for EN on HFNC. The primary outcome was time to EN initiation once on HFNC. Secondary outcomes were time to goal caloric EN, duration of HFNC, and adverse events. Outcomes were plotted on statistical process control charts and analyzed for special cause variation between baseline and intervention periods.

**Results:**

We included 112 patients in the study. Special cause variation occurred for both primary and secondary outcomes. The mean time to EN initiation decreased from 24.6 hours to 11.7 hours (47.5%). Mean time to goal feeds decreased from 25.8 hours to 15.1 hours (58.5%). Mean HFNC duration did not show any special cause variation. There were no episodes of aspiration.

**Conclusion:**

Implementation of a standardized pathway for EN on patients receiving HFNC resulted in decreased time to initiation of EN and time to goal caloric EN with no significant increase in adverse events.

## INTRODUCTION

Enteral nutrition (EN) in critically ill children has important benefits such as decreased hospital length of stay (LOS), reduced ventilator days, and fewer hospital-acquired infections.^1–4^ The stress of critical illness can cause metabolic derangements and changes in caloric requirements, increasing the risk for loss of muscle mass and body weight.^1,4^ Adult patients receiving HFNC are often underfed both in calories and in macronutrients.^5,6^ Subsequent malnutrition can lead to poor wound healing and prolonged weakness, contributing to difficulties in weaning respiratory support.^1-5^ Implementing an EN algorithm in critically ill patients decreases the time to goal calorie EN and decreases the use of parenteral nutrition.^1,3,7,8^

The most common admission diagnosis to the pediatric intensive care unit (PICU) is respiratory failure,^9^ and many patients receive support with High Flow Nasal Cannula (HFNC).^10–12^ Many pediatric centers utilize management pathways for HFNC. Yet, no standardized approach exists to initiate, escalate, and maintain EN for these patients, thus leaving room for substantial practice variability.^13,14^ Some institutional protocols include criteria for time to start EN on patients receiving HFNC treatment, and other hospitals rely solely on the service team’s clinical judgment.^13–22^ Recent studies have evaluated the safety and benefits of EN for patients receiving HFNC treatment.^8,13–22^ Various studies describe a 0–5.8% risk of pulmonary aspiration for patients fed while receiving HFNC, yet did not stratify by the method of EN delivery nor screen patients for aspiration risk.^15,21–23^ The literature also reports patients fed while receiving HFNC had decreased PICU LOS, earlier oral feeding, and received higher liter/min flow without increased adverse events.^22,23^

An international survey revealed significant variation in PICU nutrition due to a lack of standardized nutrition goals and guidelines.^24^ At our institution, some providers maintain patients receiving HFNC on dextrose-containing intravenous fluids, while others are quick to place nasogastric tubes (NGT) and feed soon after HFNC initiation. Due to the known benefits of EN in critical illness, the frequency of HFNC use, and the extensive practice variability in starting EN, we aimed to decrease the mean time to EN initiation for critically ill children in our PICU.

## METHODS

### Context

We conducted this quality improvement (QI) initiative from September 1, 2019 to March 31, 2020 at a large tertiary care urban children’s hospital with a 26-bed PICU. The patient care team included: PICU attendings and fellows, PICU hospitalists, residents, nurses, and respiratory therapists (RTs). Two pediatric certified dieticians evaluate and follow all patients receiving tube feeds. Multidisciplinary rounds occur twice daily, once on each morning and evening shifts. A pre-study survey of PICU attendings revealed the time to EN initiation was based on provider preference with advancement guided by the anticipated risk of patient aspiration or worsening respiratory failure.

At our institution, HFNC is used on the general floors and the PICU. Previously healthy patients under two years with bronchiolitis can receive HFNC up to 1.5L/kg/min on the floor. All other patients requiring HFNC are treated in the PICU. Respiratory scores (RS), adapted from Seattle Children’s Hospital,^25^ are used to titrate support for respiratory failure patients and incorporated into established bronchiolitis and status asthmaticus pathways. Our hospital utilizes Optiflow with Neotech RAM Cannula prongs and Airvo with Fisher & Paykel Optiflow Junior prongs. Our institution’s Institutional Review Board approved this QI project.

### Planning the Intervention

**Fig. 1.**
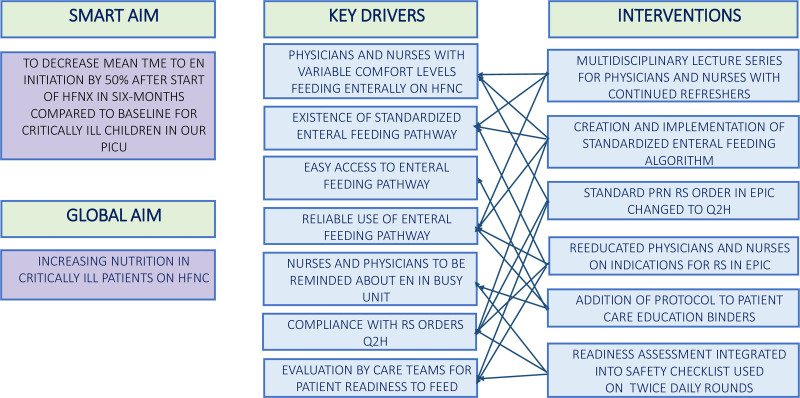
Key driver diagram for initiation of enteral nutrition for patients on HFNC.

The project aim was to decrease the mean time to EN initiation by 50% after the start of HFNC within six months compared to baseline for critically ill children in our PICU. Our intra-professional QI team included PICU attendings, a PICU fellow, resident physicians, nursing educators, charge nurses, RTs, and a dietician. The team brainstormed the root causes of delay and identified key drivers (Fig. 1) to develop interventions focused on EN pathway implementation and used the Model for Improvement (MFI) to help achieve the project aim.^26^

#### Pathway Development

**Fig. 2.**
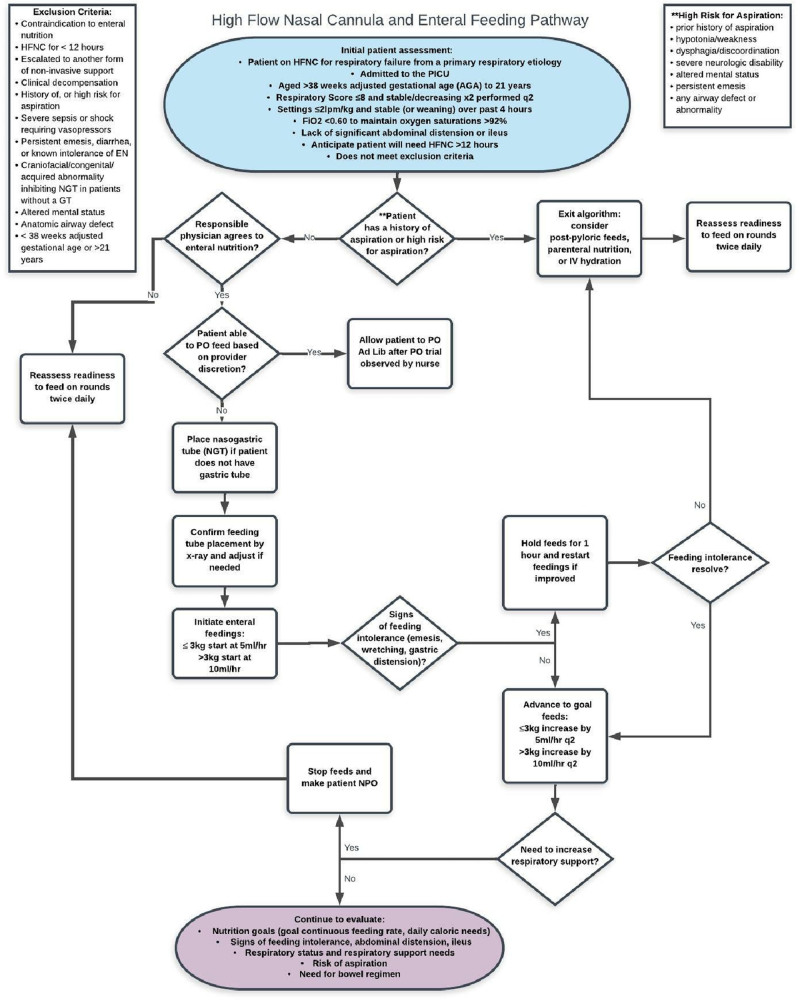
High flow nasal cannula and enteral feeding pathway.

The EN pathway (Fig. 2) begins with an initial provider assessment for every patient on HFNC, which occurs when the patient is on ≤ 2 lpm/kg of flow and stable or weaning over the previous four hours, and had a RS of <8, which was stable or decreasing after two consecutive scores (RS ordered on admission and every two hours). If the patient does not meet any exclusion criteria, the pathway has the provider evaluate if the patient was at high-risk for aspiration, defined as prior aspiration history, hypotonia/weakness, dysphagia/discoordination, severe neurologic disability, altered mental status (included patients with iatrogenic altered mental status fom medications); persistent emesis, or any airway defect or abnormality.^27^ Due to the increased risk of feeding these high-risk patients, the intervention was focused on patients at low risk for aspiration using strict inclusion and exclusion criteria. All patients at low risk for aspiration were eligible to enter the algorithm. Patients who received HFNC for less than 12 hours were not included in the analysis.

Patients who were clinically decompensating or had escalating HFNC settings and RS ≥ 9 were included but not fed until they met inclusion criteria, resulting in longer times to EN initiation from HFNC start. Due to variability in provider comfort regarding oral feeds on HFNC, the pathway suggests initiating EN via continuous tube feeding. However, based on the service team’s discretion, a patient could immediately start oral feeds, and an NGT would not be placed. These patients were not included in data analysis.

Following the initiation of the QI initiative, the QI team met weekly to discuss the pathway, data, and provider feedback and make small tests of change to address individual processes hypothesized to contribute to delay. A summary of our interventions is shown in **Supplemental Table 1**, http://links.lww.com/PQ9/A422.

### Intervention

#### Initial Education

The QI team created educational tools (Powerpoint slides, oral presentations, educational emails) to increase project awareness and provide evidence-based information and rationale for pathway development and implementation. Education to all PICU physicians, nurses, and RTs included lectures, bedside teaching, and teaching on rounds. PICU physicians who participated in pathway development, as well as nurse educators, executed the education. Due to frequent resident rotation, this education was incorporated into monthly resident PICU orientation.

#### Monitoring of Respiratory Scores Every Two Hours

Baseline data revealed RS was being ordered as PRN and recorded when calculated. Since the pathway utilizes RS as an objective measure to help safely start and continue feeds, this PDSA cycle focused on entering a standing order for nurses to complete RS every 2 hours. Physicians and nurse practioners entered orders at the time of HFNC initiation, which then appeared on nursing worklists for completion. Each completed RS was documented in the patient’s electronic health record (EHR), communicated verbally to the physician team, and discussed on rounds.

#### Pathway Implementation

After RS were consistently being ordered and recorded every 2 hours, the formal EN pathway implementation began. The pathway was given to all care team members, uploaded to the hospital intranet, placed in reference binders outside each patient’s room, and placed on the residents’ mobile workstations to facilitate patient evaluation on rounds. Care team compliance with the pathway was assessed weekly by the QI team by identifying those patients on HFNC in the PICU during the desired time frame by running an EHR report, and then comparing orders to the documentation of RS. We used RS as a surrogate because a patient could not be enrolled in the pathway without having them ordered and documented appropriately, and it was easily trackable via EHR. Patients not on the pathway had RS ordered PRN or every four hours. We learned RS orders and pathway adherence were more successful on day shifts than at night.

#### Targeted Evening Nursing Education

Targeted night shift education was provided by PICU charge nurses and nurse educators to night shift nurses, residents, fellows, and on call attendings. Education included group lectures and individualized bedside EN pathway review, focusing on the risks and benefits of EN while on HFNC.

#### Standardized Readiness Evaluation

To increase consistency of RS orders and pathway initiation, patient eligibility was assessed during both morning rounds and evening rounds. The pathway was modified to reflect this twice daily evaluation. Additionally, these items were placed on the rounding safety checklist used after each patient presentation during morning and evening rounds.

## STUDY OF THE INTERVENTION

### Measures

The primary outcome measure was time to initiation of EN once a patient began receiving HFNC. This was identified by EHR documentation indicating feeds were started. To reliably compare timing to feeds, we defined time zero as the time a patient began receiving HFNC or time of arrival to the PICU if already on HFNC. The secondary outcomes were time to achieve full caloric goals, duration of HFNC, and percentage of enrolled patients with adverse events (defined as aspiration events or need for increased respiratory support). Full caloric EN was determined by dietician evaluation of each individual patient. The process measure was the percentage of patients who had an order for RS every two hours (not PRN), as all patients receiving HFNC should have this order placed. The balancing measure was the number of patients with an NGT placed to understand if EN pathway implementation led to more patients utilizing this feeding modality.

### Evaluation and Statistical Analysis

Primary and secondary outcomes were plotted on X-bar and R statistical process control charts and analyzed for special cause variation.^26^ Control charts were created using QIMacros software (KnowWare International, Inc., Denver, Colo.) for Microsoft Excel (Microsoft, Redmond, Wa.) and annotated with PDSA cycles.

**Fig. 3.**
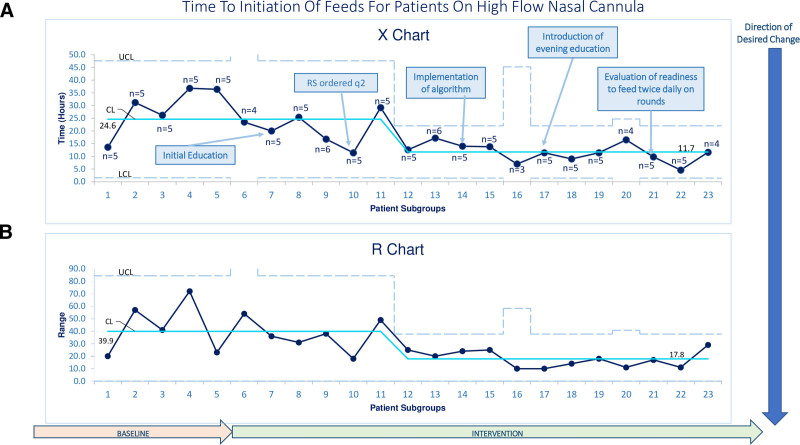
Control chart of mean time to initiation of enteral feeds for patients on high flow.

**Fig. 4.**
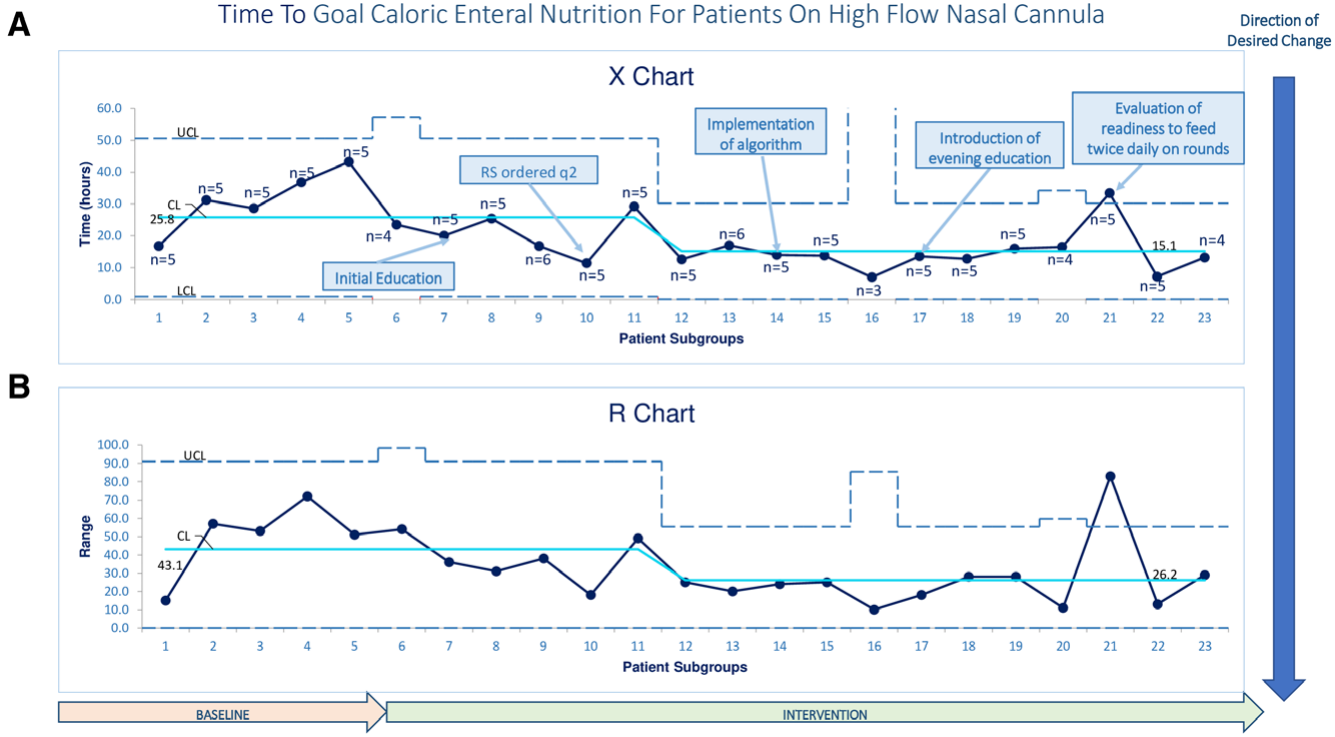
Control chart of mean time to goal caloric feeds for patients on high flow nasal cannula.

**Fig. 5.**
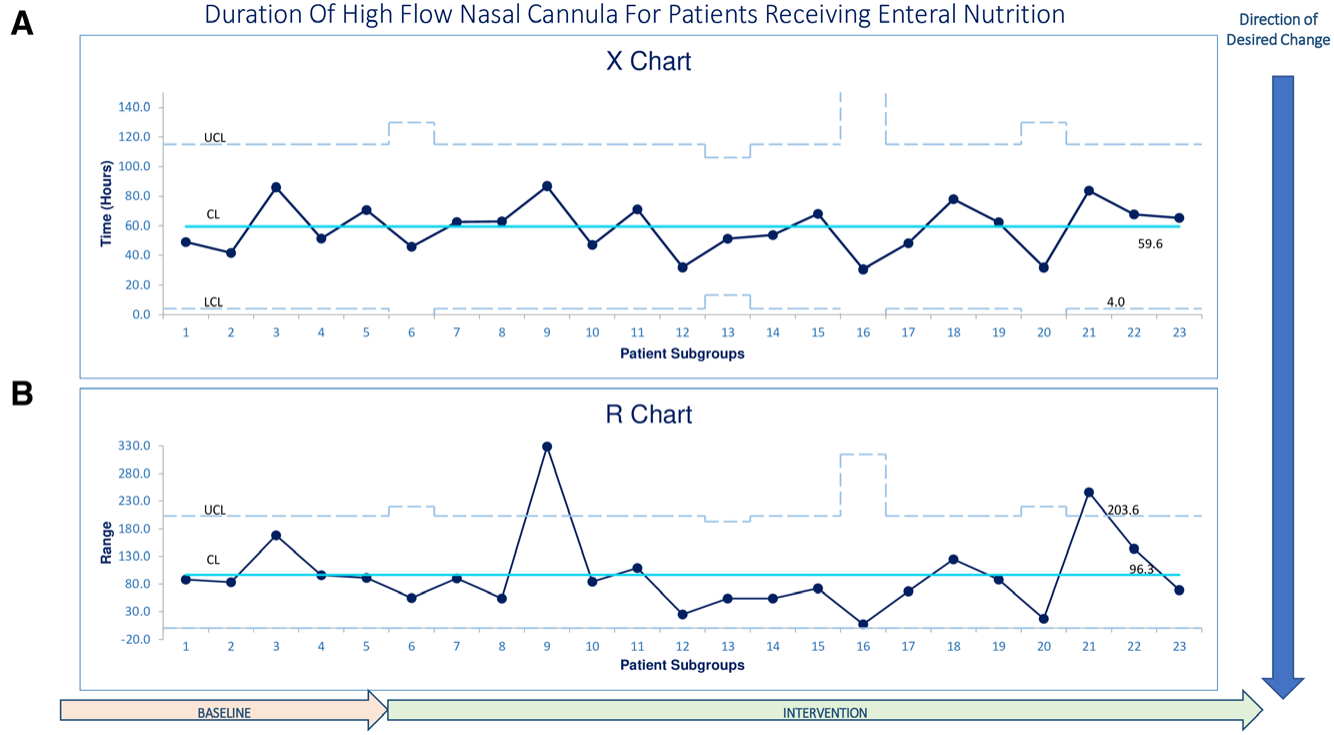
Control chart of mean duration of high flow nasal cannula.

As displayed on Figures 3–5, each data point represents the average time to the outcome for a consecutive group of patients. Groups only contained consecutive patients within the baseline period or within the indicated PDSA cycles, and groups did not include patients that crossed cycles. Most groups have five patients, except data point 6 represents the last data point in the baseline period, and points 10, 14, 17, and 23 represent the last data points in each PDSA cycle. These data points represent the average number of patients in the indicated subgroup and are between three and six patients.

Demographic data and other continuous variables were described as medians. Differences in medians between baseline and intervention data were calculated using the Mann-Whitney U test. The adverse event rate was calculated for both the baseline and intervention patients by adding number of patients with aspiration events or need for increased respiratory support over the total patients. Differences between baseline and intervention data for process and balancing measures were analyzed using chi square tests. A two-tailed *P* value < 0.05 was used to denote statistical significance.

## RESULTS

During the study period, 159 patients receiving HFNC were admitted to the PICU; 112 patients were included and 49 met exclusion criteria. Of the included patients, 29 were from the baseline period (September 1–October 17, 2019) and 83 from the intervention period (October 18, 2019–March 21, 2020). Demographic data and clinical characteristics are presented in Table 1. The baseline period had more patients with asthma (*P* = 0.017), and fewer patients with bronchiolitis (*P* = 0.042). The baseline period had a median age of 22 months compared to 18 months in the intervention period, but this was not significant (*P* = 0.07).

**Table 1.** Characteristics of the Study Population

Demographic Information	Baseline Data (n = 29)	Post-Intervention Data (n = 83)	*P*
Age in months (median)	22	18	0.07
Gender n (%)			
Men	16 (55.2%)	56 (67.5%)	0.23
Women	13 (44.8%)	27 (32.5%)	
Prior Medical History n (%)			
None	17 (58.6%)	40 (48.2%)	0.33
RAD/Asthma	4 (13.8%)	12 (14.5%)	0.93
Prematurity/BPD/CLD	3 (10.3%)	12 (14.5%)	0.58
Upper airway pathology	1 (3.4%)	3 (3.6%)	0.97
Non-cyanotic CHD	2 (6.9%)	8 (9.6%)	0.66
Seizures/neurologic disorder	3 (10.3%)	10 (12.0%)	0.81
GERD/feeding issues	2 (6.9%)	3 (3.6%)	0.46
G-Tube dependent n (%)	1 (3.4%)	4 (4.8%)	
Diagnosis n (%)			
Asthma	10 (34.5%)	10 (12.1%)	0.017
Bronchiolitis	15 (51.8%)	60 (72.3%)	0.042
Pneumonia	1 (3.4%)	9 (10.8%)	0.23
Other	3 (10.3%)	4 (4.8%)	0.29

Abbreviations: RAD, reactive airway disease; BPD, bronchopulmonary dysplasia; CLD, chronic lung disease; CHD, congenital heart disease; GERD, gastroesophageal reflux disease; G-tube, gastrostomy tube.

Figure 3 displays the time to EN initiation for all patients. Data point 12 started a shift centerline shift below the previous baseline, which continued through the rest of our data, demonstrating a 47.5% decrease in mean time to EN initiation from 24.6 hours to 11.7 hours. There was also a shift for data points 12–20 in the geSPC chart demonstrating time to goal caloric feeds (Fig. 4), showing a 58.5% mean decrease from 25.8 hours to 15.1 hours. Subgroup 21 was an astronomical data point. Mean HFNC duration is displayed in Figure 5 and did not show special cause variation.

The percent of patients who had an RS order placed every two hours, increased from 34.5% (10 of 29 patients) to 71.1% (59 of 83 patients) (*P* < 0.001). NGT placement increased from 13.8% (4 of 29) to 44.6% (37 of 83 patients) (*P* = 0.003). A gastrostomy tube was present in 3.4% of the baseline patients (1 of 29) and 4.8% of intervention patients (4 of 83) (*P* = 0.76). Among all patients, 82.8% of baseline patients and 50.6% of intervention patients were fed orally (*P* = 0.02). In the intervention period, one patient stopped feeds due to worsening respiratory failure. One patient in the baseline period and two patients in the intervention period did not tolerate feeding orally due to coughing but tolerated NGT feeds. There were no episodes of aspiration. This corresponds to a 2.4% (2 of 83) intervention adverse event rate compared to 3.5% (1 of 29) in the baseline period (*P* = 0.76).

## DISCUSSION

Implementation of a standardized EN pathway for critically ill patients on HFNC resulted in 47.5% decrease in time to initiation of EN and 58.5% decrease in time to goal caloric feeds. There was no significant difference in adverse events such as aspiration or escalated respiratory support. The shift in our data for both EN initiation and time to goal EN started in PDSA 2 while RS were being ordered every two hours, but continued and was sustained with implementation of our pathway (PDSA 3). Having the consistent and objective RS data indicating stable or improving respiratory symptoms may have prompted physicians to start feeds earlier than when RS scoring was less frequently documented. We believe this why we saw improvement in our outcomes before full pathway implementation. Data point 21 for time to goal EN was an astronomically high point, which we believe represents a reluctance by that weeks service provider to escalate feeding per the pathway.

Demographic characteristics were similar with the exceptions of more patients with asthma, and fewer patients with bronchiolitis in the baseline period. The smaller baseline sample size may account for these differences. Collection of baseline data before winter, when respiratory viruses are more prevalent, may also have contributed.

To our knowledge this is the first study reporting a detailed independent EN pathway for critically ill patients receiving HFNC. Others have incorporated EN recommendations into HFNC management pathways. A protocol-driven randomized control trial comparing NGT and NDT feeds for patients on HFNC for bronchiolitis is ongoing, but data is not yet available.^28^ Our intervention adverse event rate was 2.4%, which was not statistically different from baseline. This is lower than aspiration events reported by Slain et al. (5.8%, 70 patients),^15^ comparable to the aspiration risk in the European (325 patients) and Shadman studies (78 patients),^21-22^ and slightly higher than Conway et al (86 patients).^23^ The one patient in our cohort who required escalation of respiratory support post EN initiation was a three-year-old admitted for status asthmaticus with worsening bronchospasm while asleep requiring BiPAP. Since only nocturnal BiPAP was required, it is hard to associate this escalation with EN alone.

Our time to EN initiation is slightly longer than the four hours reported by a recent retrospective European study that evaluated the time PICU patients on non-invasive support were NPO.^21^ Most patients received continuous (48.4%), gastric (77.8%) feeds, like our population.^21^ However, Conway et al. added feeding initiation parameters to their hospital bronchiolitis pathway and reported a one-day NPO period post guideline initiation.^23^ Since our time to feed initiation was between that reported by these prior studies without an increase in adverse events, we may consider amending our pathway to have a more rapid EN up-titration.

Prior studies reported a decrease in HFNC duration associated with early EN,^4–12^ yet we showed no special cause variation, and the Conway study reported an increase in duration.^23^ Our intervention data was collected during winter when more PICU patients have respiratory failure from viral illnesses; as such, these patients may have a prolonged illness course compared to those on HFNC in the fall for other etiologies. If we had collected both phases of data in the same season, HFNC duration might have been more comparable to prior studies. We hypothesized RS would be documented more consistently, and since weaning pathways incorporate RS, patients would be weaned off HFNC faster. Our inability to reproduce these findings suggests that our clinical judgment is similarly conservative to RS in titrating support. We did not evaluate PICU/hospital LOS as patients often have longer PICU LOS during the winter due to limited bed capacity on the general floors.

Our balancing measure was NGT placement. We anticipated NGT placement wouldincrease as most baseline patients were fed orally, and the pathway suggested initiating EN by tube. Since we saw an increase in NGTs and an associated decrease in time to EN initiation and intervention goals, it may suggest providers were more comfortable feeding patients receiving HFNC by NGT earlier than they would orally. Additionally, since no appreciable increase in adverse events were seen, it may be prudent to have more concrete criteria regarding when to initiate EN orally.

For approximately three months of data collection, the PICU was full, and many patients eligible for PICU level care were managed on the general care unit supervised by intensivists. Although a large portion of these patients met criteria, we did not include them as they were managed by floor nurses who did not receive pre-intervention education and had higher nursing to patient ratios. Lastly, after 30 weeks of data collection our hospital became overwhelmed by the COVID-19 pandemic and our unit shifted to caring for COVID-19 infected adults. We excluded patients with COVID-19 given limited knowledge about the trajectory of disease.

## CONCLUSIONS

An EN pathway for patients receiving HFNC was associated with a decrease in time to initiation of EN and time to goal caloric feeds without an increase in adverse events or HFNC duration. Using a multidisciplinary approach, we achieved our aim and made a culture change in our unit. Further studies are needed to evaluate standardized EN for patients on HFNC outside the ICU setting.

## DISCLOSURE

The authors have no conflicts of interest to declare in relation to the content of this article.

## Supplementary Material


