# Supplementary material for: Feeding Pathway for Children on High Flow Nasal Cannula Decreases Time to Enteral Nutrition
Source: Pediatr Qual Saf. 2022 Dec 7;7(6):e608. doi: 10.1097/pq9.0000000000000608 (PMC9742081; doi:10.1097/pq9.0000000000000608)
Supplement: Supplementary file 1 [file pqs-7-e608-s001.pdf]

Supplemental Table 1: Summary of Interventions

Intervention	Date(s) Performed
Baseline Data Collection	September 1, 2019 – October 16, 2019
PDSA 1: Initial Education	October 17, 2019 – October 30, 2019
PDSA 2: Respiratory Score order placed in EPIC by physician	October 31, 2019 – November 21, 2019
PDSA 3: Introduction of standardized feeding algorithm	November 22, 2019 – January 3, 2020
PDSA 4: Repeat education with focus on night shift	January 4, 2020 – February 24, 2020
PDSA 5: Twice daily evaluation of patient readiness to feed on rounds	February 25, 2020 – March 31, 2020
